# Phase-Amplitude Response Functions for Transient-State Stimuli

**DOI:** 10.1186/2190-8567-3-13

**Published:** 2013-08-14

**Authors:** Oriol Castejón, Antoni Guillamon

**Affiliations:** 1 Dept. de Matemàtica Aplicada I, Universitat Politècnica de Catalunya, Avda. Diagonal 647 (ETSEIB), E-08028, Barcelona, Spain; 2 Dept. de Matemàtica Aplicada I, Universitat Politècnica de Catalunya, Dr. Marañón 44-50 (EPSEB), E-08028, Barcelona, Spain; 3 Courant Institute of Mathematical Sciences, New York University, 251 Mercer St., New York, NY, 10012-1185, USA

## Abstract

**Abstract:**

The phase response curve (PRC) is a powerful tool to study the effect of a perturbation on the phase of an oscillator, assuming that all the dynamics can be explained by the phase variable. However, factors like the rate of convergence to the oscillator, strong forcing or high stimulation frequency may invalidate the above assumption and raise the question of how is the phase variation away from an attractor. The concept of isochrons turns out to be crucial to answer this question; from it, we have built up *Phase Response Functions* (PRF) and, in the present paper, we complete the extension of advancement functions to the transient states by defining the *Amplitude Response Function* (ARF) to control changes in the *transversal variables*. Based on the knowledge of both the PRF and the ARF, we study the case of a pulse-train stimulus, and compare the predictions given by the PRC-approach (a 1D map) to those given by the PRF-ARF-approach (a 2D map); we observe differences up to two orders of magnitude in favor of the 2D predictions, especially when the stimulation frequency is high or the strength of the stimulus is large. We also explore the role of hyperbolicity of the limit cycle as well as geometric aspects of the isochrons. Summing up, we aim at enlightening the contribution of transient effects in predicting the phase response and showing the limits of the phase reduction approach to prevent from falling into wrong predictions in synchronization problems.

**List of Abbreviations:**

PRC phase response curve, phase resetting curve.

PRF phase response function.

ARF amplitude response function.

## 1 Introduction

The phase response (or *resetting*) curve (PRC) is frequently used in neuroscience to study the effect of a perturbation on the phase of a neuron with oscillatory dynamics (see surveys in [1-3]). For it to be applied, several conditions are required (weak perturbations, long time between stimuli, strong convergence to the limit cycle, etc.) so that the system relaxes back to the limit cycle before the next perturbation/kick is received. In this case, one can reduce the study to the phase dynamics on the oscillatory solution (namely, a limit cycle). However, in realistic situations, we may not be able to determine whether the system is on an attractor (limit cycle); moreover, the system may not show regular spiking, especially because of noise; see for instance [4,5]. In addition, even in the absence of noise, strong forcing may send the dynamics away from the asymptotic state, eventually close to other nearby invariant manifolds [6]; thus, both the rate of convergence to the attractor and the stimulation frequency (which can be relatively high; take for instance the case of bursting-like stimuli) play an important role in controlling the time spent in the transient state (away from the limit cycle). All these factors may prevent the trajectories from relaxing back to the limit cycle before the next stimulus arrives and raise the question of the nature of the phase variation away from an attractor (that is, in transient states) and how much can we rely on the phase reduction (PRC). 

Recently, tools to study the phase variation away from a limit cycle attractor have been developed. They rely on the concept of isochrons (manifolds transversal to the limit cycle and invariant under time maps given by the flow), introduced by Winfree (see [7]) in biological problems, from which one can extend the definition of phase in a neighborhood of the limit cycle. In a previous paper [8,9], we showed how to compute a parameterization of the isochrons (see also [10-12] for other computational methods) as well as the change in phase due to the kicks received when the system is approaching the limit cycle but not yet on it. This approach allowed us to control the phase advancement away from the limit cycle (that is, in the transient states) and build up the *Phase Response Functions* (PRF), a generalization of the PRCs. In [8], examples of neuron oscillators were shown in which the phase advancement was clearly different for states sharing the same phase. A review of these tools is presented in Sect. 2.

In Sect. 3, we complete the extension of advancement functions to the transient states by defining the *Amplitude Response Function* (ARF), and we provide methods to compute it by controlling the changes induced by perturbations in a *transversal variable*, which represents some distance to the limit cycle. One of the methods presented here to compute the ARFs is an extension of the well-known *adjoint method* for the computation of PRCs; see, for instance, [13,14] or Chap. 10 in [1]. 

Indeed, the knowledge of both the PRF and the ARF allows us to consider special problems in which these functions can forecast the asymptotic phase of an oscillator under pulsatile repetitive stimuli. In the case of a pulse-train stimulus, the variations of the extended phase and the amplitude can be controlled by means of a 2D map; this 2D map extends the classical 1D map used when the dynamics is restricted to the limit cycle or phase-reduction is assumed; see, for instance Chap. 10 in [1]. Another successful strategy to deal with kicks that send the dynamics away from the limit cycle is the so-called second-order PRC (see [15-17]), which measures the effects of the kick on the next cycle period, taking into account that synaptic input can span two cycles. 

As an illustration of the method, in Sect. 4, we then consider a canonical model for which we compute the PRFs and ARFs thanks to the exact knowledge of the isochrons. In this “canonical” example, we apply a two parametric periodic forcing (varying the stimulus strength and frequency) and make predictions both with our 2D map and the classical 1D map; we use rotation numbers to illustrate the differences between the two predictions and we observe differences up to two orders of magnitude in favor of the 2D predictions, especially when the stimulation frequency is high or the strength of the stimulus is large. We also use this example to shed light on the role of hyperbolicity of the limit cycle as well as geometric aspects of the isochrons (see also [18] for a related study of the effect of isochrons’ shear). Finally, we also present the comparison of the two approaches in a conductance-based neuron model, where we do not know the isochrons analytically. 

Summing up, we aim at enlightening the contribution of transient effects in predicting the phase response, focusing on the importance of the “degree” of hyperbolicity of the limit cycle, but also on the relative positions of the isochrons with respect to the limit cycle. Since PRCs are used for predicting synchronization properties, see [19], Chap. 10 in [1] or Chap. 8 in [2], our final goal is to show the limits of the phase reduction approach to prevent wrong predictions in synchronization problems. 

## 2 Set-up of the Problem: Isochrons and Phase Response Functions (PRF)

In this section, we set up the problem and we review some of the results in [8] that serve as a starting point of the study that we present in this paper. 

Consider an autonomous system of ODEs in the plane

(1) $$\dot{x}=X(x),\;x\in U\subseteq R^{2},$$

and denote by $\phi_{t}$ the flow associated to (1). Assume that *X* is an analytic vector field and that (1) has a hyperbolic limit cycle *Γ* of period *T*, parameterized by θ=t/T as

(2) $$\begin{array}{rl} \gamma :T & \to R^{2}\\ \theta & \mapsto \gamma (\theta ), \end{array}$$

where T=R/Z, so that γ(θ)=γ(θ+1).

Under these conditions, by the stable manifold theorem (see [20]), there exists a unique scalar function defined in a neighborhood *Ω* of the limit cycle *Γ*,

(3) $$\begin{array}{rl} \Theta :\Omega \subset R^{2} & \to T\\ x & \mapsto \Theta (x) \end{array}$$

such that

(4) $$\lim_{t\to +\infty }\left|\phi_{t}(x)-\gamma \left(t/T+\Theta (x)\right)\right|=0,$$

if the limit cycle is attracting. If the limit cycle is repelling, the same is true with t→−∞.

The value Θ(x) is the asymptotic phase of **x** and the isochrons are defined as the sets with constant asymptotic phase, that is, the level sets of the function *Θ*. The same construction can be extended to limit cycles in higher dimensional spaces, but since the applications in this paper will be restricted to planar systems, we give the definitions in $R^{2}$.

Moreover, we know from [21] that there exists an analytic local diffeomorphism

(5) $$\begin{array}{rl} K:T\times [\sigma_{-},\sigma_{+}] & \to R^{2}\\ \left(\theta ,\sigma \right) & \mapsto K(\theta ,\sigma ), \end{array}$$

satisfying the invariance equation

(6) $$\left(\frac{1}{T}\partial_{\theta }+\frac{\lambda \sigma }{T}\partial_{\sigma }\right)K(\theta ,\sigma )=X\left(K(\theta ,\sigma )\right),$$

where *T* is the period and *λ* is the characteristic exponent of the periodic orbit.

**Fig. 1:**
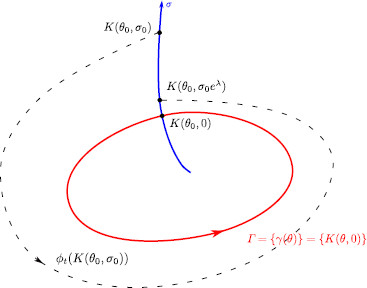
The new coordinate system. Representation of a limit cycle (*red*) and an isochron (*blue*) corresponding to the phase level sets $\theta =\theta_{0}$. *The black dashed curve* is a piece of the trajectory of the vector field *X* starting at a generic point $K(\theta_{0},\sigma_{0})$

We can describe (6) as saying that if we perform the change of variables given by *K*, the dynamics of the system (1) in the coordinates (θ,σ) consist of a rigid rotation with constant velocity 1/T for *θ* and a contraction (if λ<0) with exponential rate λ/T for *σ*. That is,

(7) $$\begin{array}{rl} \dot{\theta } & =1/T,\\ \dot{\sigma } & =\lambda \sigma /T, \end{array}$$

and $\phi_{t}(K(\theta ,\sigma ))=K(\theta +t/T,\sigma e^{(\lambda /T)t})$, where $\phi_{t}$ is the flow associated to (1). See Fig. 1 for an illustration of the evolution of the variables (θ,σ) along an orbit of the system. 

*Remark 2.1* In [8], we presented a computational method to compute the parameterization *K* defined in (6) numerically.

Given an analytic local diffeomorphism *K* satisfying (6), we know from Theorem 3.1 in [8] that the isochrons are the orbits of a vector field *Y* satisfying the Lie symmetry [Y,X]=μY with μ=λ/T. That is,

(8) $$Y\circ K(\theta ,\sigma )=\partial_{\sigma }K(\theta ,\sigma ).$$

Let us assume that a pulse of small modulus *ϵ* instantaneously displaces the trajectory in a direction given by the unit vector **w**. Mathematically, we consider

(9) $$\dot{x}=X(x)+\epsilon w\delta (t-t_{s}),$$

where ϵ≪1 and δ(t) is the Dirac delta function. Then we define the phase response function (PRF) as the infinitesimal rate of change of the phase in the direction **w** of the perturbation, that is,

$$\mathrm{PRF}(x)=D_{w}\Theta (x)=\langle w,\nabla \Theta (x)\rangle ,$$

where *Θ* is the phase function defined in (3) and 〈⋅,⋅〉 denotes the dot product. In [8], we showed that

(10) $$\nabla \Theta \left(K(\theta ,\sigma )\right)=\frac{{\left(\partial_{\sigma }K\right)}^{⊥}}{T\langle {\left(\partial_{\sigma }K\right)}^{⊥},\partial_{\theta }K\rangle },$$

where ${\left(\partial_{\sigma }K\right)}^{⊥}=J(\partial_{\sigma }K)$ and the matrix *J* is given by

(11) $$J=\left(\begin{array}{cc} 0 & -1\\ 1 & 0 \end{array}\right).$$

We will use this notation for the rest of the paper.

In neuron models, the usual situation is x=(V,…) and w=(1,0,…,0); thus, the phase response function (PRF) is defined as

(12) $$\mathrm{PRF}(x)=\partial_{V}\Theta (x),$$

where $\partial_{V}$ denotes partial derivative with respect to the variable *V*.

## 3 The Amplitude Response Function (ARF)

A pulse stimulation displaces the trajectory away from the limit cycle, producing a change both in the phase *θ* and the transversal variable *σ*, that we will refer to as the *amplitude* variable. In our notation, the amplitude variable is a distance measure defined by the time from the limit cycle along the orbits of the auxiliary vector field *Y*, transversal to *X*, defined in (8). In fact, the orbits of the vector field *Y* are the isochrons; see, for instance, the blue curve in Fig. 1. The phase-reduction approach assumes that the amplitude decreases to zero before the next pulse arrives and, therefore, the amplitude is always zero at the stimulation time. However, if one wants to consider pulses that arrive before the trajectory relaxes back to the limit cycle, one needs to compute also the amplitude displacement in order to predict the coordinates of the point at the next stimulation time.

In this section, we introduce the amplitude function and Amplitude Response Function (ARF), the analogues of the phase function (3) and the PRF (12) for the variable *σ*. Finally, we provide a formula to compute them given the diffeomorphism *K* introduced in (5).

### 3.1 Definitions

Given an analytic local diffeomorphism *K* as in (5) satisfying (6), it follows that there exists a unique function *Σ*, defined in a neighborhood *Ω* of the limit cycle *Γ*,

(13) $$\begin{array}{rl} \Sigma :\Omega \subset R^{2} & \to R\\ x & \mapsto \Sigma (x) \end{array}$$

such that

$$\Sigma \left(\phi_{t}(x)\right)=\Sigma (x)e^{\lambda t/T},$$

where $\phi_{t}$ is the flow associated to the vector field *X*. The level curves of *Σ* are closed curves that we will call *amplitude level curves* or, in short, *A-curves*.

Analogous to the phase isochrons, it can be seen that given an analytic local diffeomorphism *K*, as in (5), satisfying (6), the A-curves are the orbits of a vector field *Z*, satisfying [X,Z]=[Z,X]=0; see the Appendix for a proof of this result. More specifically,

(14) $$Z\left(K(\theta ,\sigma )\right)=\partial_{\theta }K(\theta ,\sigma ).$$

Expressed in the variables (θ,σ) introduced in (5), the motion generated by *Z* is given by $\left\{\dot{\theta }=1,\dot{\sigma }=0\right\}$.

A pulsatile kick in the direction given by the unit vector **w** (see (9)) causes a change in the amplitude variable. Analogous to the PRF introduced in (10), we define the *Amplitude Response Function* (ARF) as the infinitesimal rate of change of the phase in the direction **w** of the perturbation, that is,

$$\mathrm{ARF}(x)=D_{w}\Sigma (x)=\langle w,\nabla \Sigma (x)\rangle .$$

In neuron models, the ARF typically measures the change in amplitude under the action of a pulsatile kick in the direction of the voltage *V*, that is,

$$\mathrm{ARF}(x)=\partial_{V}\Sigma (x),$$

where $\partial_{V}$ denotes partial derivative with respect to the variable *V*.

### 3.2 Computation of the PRFs and the ARFs

In this section, we provide a formula to compute the functions ∇*Θ* and ∇*Σ* given the diffeomorphism *K* introduced in (5).

Using the parameterization *K* introduced in (5) and writing $K(x,y)=K(\Theta (x,y),\Sigma (x,y))=(K_{x},K_{y})$, where *Θ* and *Σ* are the functions introduced in (3) and (13), respectively, we have that

$$\left(\begin{array}{cc} \partial_{\theta }K_{x} & \partial_{\sigma }K_{x}\\ \partial_{\theta }K_{y} & \partial_{\sigma }K_{y} \end{array}\right)\left(\begin{array}{cc} \partial_{x}\Theta & \partial_{y}\Theta \\ \partial_{x}\Sigma & \partial_{y}\Sigma \end{array}\right)=\left(\begin{array}{cc} 1 & 0\\ 0 & 1 \end{array}\right),$$

and, therefore, $\nabla \Theta =(\partial_{x}\Theta ,\partial_{y}\Theta )$ and $\nabla \Sigma =(\partial_{x}\Sigma ,\partial_{y}\Sigma )$ are given by

$$\begin{array}{rcl} \left(\begin{array}{c} \nabla \Theta \\ \nabla \Sigma \end{array}\right) & = & {\left(\begin{array}{cc} \partial_{\theta }K_{x} & \partial_{\sigma }K_{x}\\ \partial_{\theta }K_{y} & \partial_{\sigma }K_{y} \end{array}\right)}^{-1}\\ & = & \frac{1}{\langle \partial_{\sigma }K^{⊥},\partial_{\theta }K\rangle }\left(\begin{array}{cc} \partial_{\sigma }K_{y} & -\partial_{\sigma }K_{x}\\ -\partial_{\theta }K_{y} & \partial_{\theta }K_{x} \end{array}\right)\\ & = & \frac{1}{\langle \partial_{\sigma }K^{⊥},\partial_{\theta }K\rangle }\left(\begin{array}{c} \partial_{\sigma }K^{⊥}\\ \partial_{\theta }K^{⊥} \end{array}\right). \end{array}$$

Hence,

(15) $$\begin{array}{rl} \nabla \Theta \left(K(\theta ,\sigma )\right) & =\frac{\partial_{\sigma }K^{⊥}(\theta ,\sigma )}{\langle \partial_{\sigma }K^{⊥}(\theta ,\sigma ),\partial_{\theta }K(\theta ,\sigma )\rangle },\;\text{and}\\ \nabla \Sigma \left(K(\theta ,\sigma )\right) & =\frac{\partial_{\theta }K^{⊥}(\theta ,\sigma )}{\langle \partial_{\sigma }K^{⊥}(\theta ,\sigma ),\partial_{\theta }K(\theta ,\sigma )\rangle }. \end{array}$$

Using the vector field description given in (8) and (14), we can rewrite the expression above, using the relations $\partial_{\sigma }K=Y\circ K$ and $\partial_{\theta }K=Z\circ K$, as

$$\nabla \Theta \left(K(\theta ,\sigma )\right)=\frac{Y^{⊥}}{\langle Y^{⊥},Z\rangle }{|}_{K(\theta ,\sigma )},\;\text{and}\;\nabla \Sigma \left(K(\theta ,\sigma )\right)=\frac{Z^{⊥}}{\langle Z^{⊥},Y\rangle }{|}_{K(\theta ,\sigma )}.$$

By the invariance equation (6), we know that $X=\frac{1}{T}Z+\frac{\lambda }{T}\sigma Y$ and, therefore,

(16) $$\begin{array}{rl} \nabla \Theta \left(K(\theta ,\sigma )\right) & =\frac{Y^{⊥}}{T\langle Y^{⊥},X\rangle }{|}_{K(\theta ,\sigma )},\;\text{and}\\ \nabla \Sigma \left(K(\theta ,\sigma )\right) & =\frac{\lambda \sigma }{T}\frac{Z^{⊥}}{\langle Z^{⊥},X\rangle }{|}_{K(\theta ,\sigma )}. \end{array}$$

*Remark 3.1* Notice that expression for ∇*Σ* in (16) might suggest that it has a singularity at σ=0. Nevertheless, the vanishing terms in the numerator and denominator cancel out at σ=0, and using that $Z(K(\theta ,0))=\partial_{\theta }K(\theta ,0)=X(K(\theta ,0))$, the value at σ=0 is given by

$$\nabla \Sigma \left(\gamma (\theta )\right)=\frac{X^{⊥}(\gamma (\theta ))}{\langle X^{⊥}(\gamma (\theta )),K_{1}(\theta )\rangle },$$

where $K_{1}(\theta )=\partial_{\sigma }K(\theta ,0)=Y(K(\theta ,0))$.

### 3.3 The Adjoint Method for the ARF

The most common method to compute the PRC, the so-called *adjoint method*, uses that the function ∇*Θ* evaluated on the limit cycle *Γ* is a periodic solution of some adjoint equation (see, for instance, [1]). In the generalization introduced in [8], it was shown that the adjoint method can be extended to compute ∇*Θ* for points in a neighborhood of the limit cycle, for which the periodicity condition is not satisfied. Indeed, Q=∇Θ satisfies the equation

(17) $$\frac{dQ}{dt}=-DX^{T}\left(\phi_{t}(p)\right)Q,$$

where $DX^{T}$ is the transpose of the real matrix *DX*. In this case, the method just requires an initial condition, so that it can be solved uniquely. The initial condition is provided by formula (15).

The same result can be extended to compute the change in the transversal variable *σ* due to a pulse stimulation. In the following proposition, we provide the differential equation satisfied by ∇Σ(p) where p=K(θ,σ) is a point in a neighborhood *Ω* of the limit cycle *γ* evolving under the flow of *X*.

**Proposition 3.2***LetΓbe a hyperbolicT*-*periodic orbit of a planar analytic vector fieldXparameterized byθaccording to* (2). *Assume that there exists a change of coordinatesKin a neighborhoodΩsatisfying* (6). *Then the function* ∇*Σalong the orbits of the vector fieldXsatisfies the adjoint equation*

(18) $$\frac{dQ}{dt}=\left(\frac{\lambda }{T}-DX^{T}\left(\phi_{t}(p)\right)\right)Q,$$

*where*$\phi_{t}$*is the flow of the vector fieldXandλis the characteristic multiplier of the periodic orbit*, *with the initial condition*

(19) $$Q(0)=\frac{\lambda \Sigma (p)}{T}\frac{Z^{⊥}(p)}{\langle Z^{⊥}(p),X(p)\rangle },$$

*where*$Z^{⊥}(K(\theta ,\sigma ))=J\partial_{\theta }K(\theta ,\sigma )$.

*Proof* We will show that the function ∇*Σ* evaluated along the orbits $\phi_{t}(p)$ of *X* satisfies the adjoint equation (18). From expression (16), we have that

(20) $$\nabla \Sigma \left(\phi_{t}(p)\right)=\frac{\lambda \Sigma (\phi_{t}(p))}{T}\frac{Z^{⊥}(\phi_{t}(p))}{\langle Z^{⊥}(\phi_{t}(p)),X(\phi_{t}(p))\rangle }.$$

We now compute the derivative of $\nabla \Sigma (\phi_{t}(p))$ with respect to time. In order to simplify notation, we set $x:=\phi_{t}(p)$. We will also use that $Z^{⊥}=JZ$ where *J* is the matrix (11). Using that $\frac{d}{dt}Z(x)=DZ(x)X(x)$, we have from (20)

$$\begin{array}{rcl} \frac{d}{dt}\nabla \Sigma (x) & = & \frac{\lambda (d\Sigma /dt)(x)JZ(x)+\lambda \Sigma (x)JDZ(x)X(x)}{T\langle Z^{⊥}(x),X(x)\rangle }\\ & & -\frac{\lambda \Sigma (x)JZ(x)(\langle JDZ(x)X(x),X(x)\rangle +\langle JZ(x),DX(x)X(x)\rangle )}{T{\langle Z^{⊥}(x),X(x)\rangle }^{2}}. \end{array}$$

Using that DXZ=DZX, expression (20) and dot product properties (namely, $\langle JZ(x),DX(x)X(x)\rangle =\langle DX{\left(x\right)}^{T}JZ(x),X(x)\rangle$), we obtain

$$\begin{array}{rcl} \frac{d}{dt}\nabla \Sigma (x) & = & \frac{\left(\lambda /T)\lambda \Sigma (x)JZ(x)+\lambda \Sigma (x)JDX(x)Z(x\right)}{Tg(x)}\\ & & -\frac{\nabla \Sigma (x)\langle JDX(x)Z(x)+DX{\left(x\right)}^{T}JZ(x),X(x)\rangle }{\langle Z^{⊥}(x),X(x)\rangle }. \end{array}$$

Using $JDX(x)+DX{\left(x\right)}^{T}J=\mathrm{trace}(DX)(x)J$, and denoting τ(x):=trace(DX)(x), we are led to

$$\frac{d}{dt}\nabla \Sigma (x)=\frac{\left(\lambda /T-DX{\left(x\right)}^{T}+\tau (x))\lambda \Sigma (x)JZ(x\right)}{T\langle Z^{⊥}(x),X(x)\rangle }-\frac{\nabla \Sigma (x)(\langle \tau (x)JZ(x),X(x)\rangle )}{\langle Z^{⊥}(x),X(x)\rangle }.$$

Finally, using again (20), we have

$$\begin{array}{rcl} \frac{d}{dt}\nabla \Sigma (x) & = & \left(\lambda /T-DX{\left(x\right)}^{T}+\tau (x)\right)\nabla \Sigma (x)-\nabla \Sigma (x)\tau (x)\\ & = & \left(\lambda /T-DX{\left(x\right)}^{T}\right)\nabla \Sigma (x), \end{array}$$

as we wanted to prove. □

## 4 Periodic Pulse-Train Stimuli

The purpose of this section is to show the convenience of using the response functions away from the limit cycle to obtain accurate predictions of the ultimate phase advancement. To this end, we force a system with pulse-trains of period $T_{s}\ll T_{0}$ for trajectories near a limit cycle *Γ* of period $T_{0}$ and characteristic exponent *λ*.

Given an oscillator, assume that it is perturbed with an external instantaneous stimulus of amplitude *ϵ* in the voltage direction every $T_{s}$ time units, that is,

(21) $$\dot{x}=X(x)+\epsilon w\sum_{j=0}^{N}\delta (t-jT_{s}),$$

where w=(1,0), ϵ≪1 and *δ* is the Dirac delta function. This system can represent, for example, a neuron which receives an idealized synaptic input from other neurons.

*Remark 4.1* In the sequel, we will also use $\omega_{s}=1/T_{s}$, the frequency of the stimulus, and $\omega_{0}=1/T_{0}$, the frequency of the limit cycle *Γ*. Then the quotient $\omega_{s}/\omega_{0}$ indicates how many inputs the oscillator receives in one period.

In order to know the evolution of the perturbed oscillator after each time period $T_{s}$, it is enough to know how the variables *θ* and *σ* change. We recall that the variation of the variable *θ* produced by an external stimulus is given, to first order in the stimulus strength *ϵ*, by the PRF. Similarly, the variation of the variable *σ* is given to first order by the ARF. Hence, we can consider the following map, which approximates the position of the oscillator at the moment of the next kick:

(22) $$\begin{array}{rl} \theta_{n+1} & =\theta_{n}+\epsilon \mathrm{PRF}(\theta_{n},\sigma_{n})+\frac{T_{s}}{T_{0}}\;(\mathrm{mod}1),\\ \sigma_{n+1} & =\left(\sigma_{n}+\epsilon \mathrm{ARF}(\theta_{n},\sigma_{n})\right)e^{\lambda T_{s}/T_{0}}. \end{array}$$

Moreover, we can compare it with the map obtained by considering the classical PRC (see, for instance, Chap. 10 in [1]), which is

(23) $$\theta_{n+1}=\theta_{n}+\epsilon \mathrm{PRC}(\theta_{n})+\frac{T_{s}}{T_{0}}\;(\mathrm{mod}1).$$

In the latter case, we are assuming that the perturbation happens always on the limit cycle and, therefore, $\sigma_{n}=0$ for all *n*. The possibility that this might not be a realistic assumption (for example, if the stimulus period $T_{s}$ is too small, the limit cycle is weakly hyperbolic or the strength of the stimulus *ϵ* is too large) has been already pointed out in the literature; see, for instance, [22] or Chap. 10 in [1]. However, other factors could play a role, for example, the geometry of the isochrons (curvature, transversality to the limit cycle, etc.). Our aim is to consider some examples and see in which cases the 1D map (23) gives a correct prediction or, on the contrary, one requires the 2D map (22) to correctly assess the true dynamics of the phase variable.

To quantify the long-term agreement or disagreement between the 1D and the 2D predictions, we compute an approximation of the rotation numbers after *N* iterations for both maps. More precisely, given an initial condition $\left(\theta_{0},\sigma_{0}\right)$, we compute

(24) $$\bar{\rho }=\lim_{N\to +\infty }\frac{1}{N}\sum_{j=1}^{N}({\tilde{\theta }}_{j}-{\tilde{\theta }}_{j-1}),$$

where $\tilde{\theta }$ denotes the lift of *θ* to ℝ. Then, for the 2-dimensional map (22) and assuming *N* large enough, the rotation number can be approximated by

(25) $$\rho_{2D}:=\frac{T_{s}}{T_{0}}+\frac{1}{N}\epsilon \sum_{j=0}^{N-1}\mathrm{PRF}(\theta_{j},\sigma_{j}),$$

and by

(26) $$\rho_{1D}:=\frac{T_{s}}{T_{0}}+\frac{1}{N}\epsilon \sum_{j=0}^{N-1}\mathrm{PRC}(\theta_{j}),$$

for the 1-dimensional map (23).

These approximate rotation numbers will be our main indicator to compare the dynamics predicted by the 1D map with that of the 2D map. In order to dissect the causes that create the eventual differences between the two maps and highlight the shortcomings of the phase-reduction approach, we have first considered a “canonical” example in which the isochrons can be computed analytically. Next, we consider a conductance-based model, in which the isochrons have to be computed numerically and we obtain similar comparative results.

### 4.1 Examples

#### 4.1.1 A Simple Canonical Model

We consider a simple model having a limit cycle with two parameters, *α* and *a*, that control the hyperbolicity of the limit cycle and the angle between the isochrons and the limit cycle, respectively. The system has the following expression in polar coordinates:

(27) $$\begin{array}{rl} \dot{r} & =\alpha r\left(1-r^{2}\right),\\ \dot{\varphi } & =1+\alpha ar^{2}, \end{array}$$

for a,α∈R, and the following one in Cartesian coordinates:

(28) $$\begin{array}{rl} \dot{x} & =\alpha x\left(1-\left(x^{2}+y^{2}\right)\right)-y\left(1+\alpha a\left(x^{2}+y^{2}\right)\right),\\ \dot{y} & =\alpha y\left(1-\left(x^{2}+y^{2}\right)\right)+x\left(1+\alpha a\left(x^{2}+y^{2}\right)\right). \end{array}$$

The limit cycle corresponds to r=1 and the dynamics on it are given by $\dot{\varphi }=1+\alpha a$; therefore, $\varphi (t)=\varphi_{0}+(1+\alpha a)t\mathrm{mod}2\pi$. The period of the limit cycle *Γ* is $T_{0}=2\pi /(1+\alpha a)$. A parameterization of the limit cycle in terms of the phase $\theta =t/T_{0}$, for θ∈[0,1) is γ(θ)=(cos(2πθ),sin(2πθ)).

Now, consider the vector field *Y*, given in the polar and Cartesian coordinates by

$$\begin{array}{lll} \dot{r}=\alpha r^{3}, & \text{and} & \dot{x}=\alpha \left(x^{2}+y^{2}\right)(x+ay),\\ \dot{\varphi }=-\alpha ar^{2}; & & \dot{y}=\alpha \left(x^{2}+y^{2}\right)(y-ax). \end{array}$$

It is easy to check that *Y* satisfies [X,Y]=−2αY. Then, using (8), we find that μ=−2α and

(29) $$\begin{array}{rcl} K(\theta ,\sigma ) & = & (\sqrt{\frac{1}{1-2\alpha \sigma }}\cos\left(2\pi \theta +\frac{1}{2}a\ln(1-2\alpha \sigma )\right),\\ & & \sqrt{\frac{1}{1-2\alpha \sigma }}\sin\left(2\pi \theta +\frac{1}{2}a\ln(1-2\alpha \sigma )\right)), \end{array}$$

with θ∈[0,1) and σ>1/(2α).

Notice that the function *K* can be easily inverted using that $r^{2}=x^{2}+y^{2}={\left(1-2\alpha \sigma \right)}^{-1}$ and $\arctan(\frac{y}{x})=2\pi \theta +\frac{1}{2}a\ln(1-2\alpha \sigma )$. Then $K^{-1}(x,y)=(\Theta (x,y),\Sigma (x,y))$, where

$$\Theta (x,y)=\frac{1}{2\pi }\left(\arctan\left(\frac{y}{x}\right)-\frac{1}{2}a\ln\left(\frac{1}{r^{2}}\right)\right),\;\Sigma (x,y)=\frac{1}{2\alpha }\left(1-\frac{1}{r^{2}}\right).$$

Thus, the dynamics for (θ,σ) are given by

(30) $$\begin{array}{rl} \dot{\theta } & =1/T_{0},\\ \dot{\sigma } & =-2\alpha \sigma . \end{array}$$

The vector field *Z*, defined in (14), has the following expression in Cartesian coordinates and polar coordinates, respectively:

$$\begin{array}{lll} \dot{x}=-2\pi y, & \text{and} & \dot{r}=0,\\ \dot{y}=2\pi x; & & \dot{\varphi }=2\pi . \end{array}$$

Therefore, we find that $\nabla \Theta (p)=\frac{1}{2\pi r^{2}}(-y+ax,x+ay)$, and, by the parameterization *γ* of the limit cycle, $\nabla \Theta (\gamma (\theta ))=\frac{1}{2\pi }(-\sin(2\pi \theta )+a\cos(2\pi \theta ),\cos(2\pi \theta )+a\sin(2\pi \theta ))$. Similarly, $\nabla \Sigma (p)=(\frac{x}{\alpha r^{4}},\frac{y}{\alpha r^{4}})$, and ∇Σ(γ(θ))=(cos(2πθ),sin(2πθ)).

From the last equations, we can then obtain

(31) $$\begin{array}{rcl} \mathrm{PRF}\left(K(\theta ,\sigma )\right) & = & -\frac{\sqrt{1-2\alpha \sigma }}{2\pi }(\sin\left(2\pi \theta +\frac{1}{2}a\ln(1-2\alpha \sigma )\right)\\ & & -a\cos\left(2\pi \theta +\frac{1}{2}a\ln(1-2\alpha \sigma )\right)) \end{array}$$

and

$$\mathrm{ARF}\left(K(\theta ,\sigma )\right)=\frac{{\left(1-2\alpha \sigma \right)}^{3/2}}{\alpha }\cos\left(2\pi \theta +\frac{1}{2}a\ln(1-2\alpha \sigma )\right).$$

**Fig. 2:**
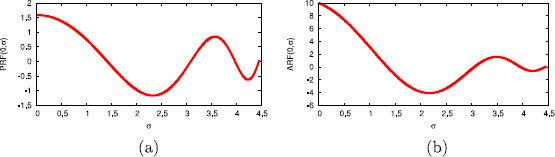
Phase and amplitude response functions along isochrons. Phase response function (PRF) and amplitude response function (ARF) for system (28) with a=10 and α=0.1, computed from (31)

In Fig. 2, we show the PRF and the ARF for a specific isochron for representative values of the parameters, a=10 and α=0.1. An important remark is that the PRF is far from being constant along isochrons, whereas the ARF is clearly nonzero near the limit cycle (σ=0). These features will have a significant effect when comparing the predictions provided by the 1D map and the 2D map. 

We want to stress the role of the parameters *α* and *a*. On one hand, the parameter *α* determines the hyperbolicity of the limit cycle, since its characteristic exponent is

$$\lambda =-2\alpha T_{0}.$$

Hence, for small values of *α* the contraction to the limit cycle will be weak, while as *α* goes to infinity *λ* tends to −4π/a. On the other hand, the parameter *a* determines the transversality of the isochrons to the limit cycle. Indeed, denoting by *β* the angle between the isochron $\left\{p\in R^{2}:\Theta (p)=\theta \right\}$ and the limit cycle at the point γ(θ), we have

$$\cos\beta =\frac{\langle \gamma^{\prime }(\theta ),\nabla \Theta^{⊥}(\gamma (\theta ))\rangle }{∥\gamma^{\prime }(\theta )∥∥\nabla \Theta^{⊥}(\gamma (\theta ))∥}.$$

Computing explicitly the right-hand side of the equality, it is straightforward to verify that

$$\cos\beta =\frac{2\pi a}{\sqrt{1+a^{2}}}.$$

**Fig. 3:**
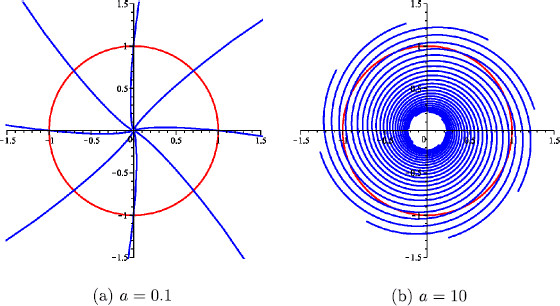
Limit cycle and isochrons. The limit cycle (*red*) of system (28) and some isochrons (*blue*) for different values of the parameter *a*. In both cases, α=10

In particular, note that *β* is independent of the variable *θ*. Moreover, for a=0 the isochrons are orthogonal to the limit cycle and, as *a* tends to infinity, they become to it (see Fig. 3). 

#### 4.1.2 Numerical Simulations

In this section, we use the analytic expressions obtained in the previous subsection for the PRF, ARF, and PRC to compute and compare the maps defined in (22) and (23). Moreover, as we also have an explicit formula for the parameterization *K* and its inverse $K^{-1}$, we can integrate numerically system (28), perturb it periodically, and obtain a sequence $\left(x_{n},y_{n}\right)$. Then we can compute analytically

(32) $$(\theta_{n},\sigma_{n})=K^{-1}(x_{n},y_{n}),$$

and compare it with the iterations obtained using the maps (22) and (23). In the following, we will call the approximation of the rotation number obtained by this method simply *ρ*, to distinguish it from $\rho_{2D}$ and $\rho_{1D}$ defined previously in (25) and (26), respectively. The following lemma gives a description of the dynamics expected in the 1-dimensional map.

**Lemma 4.2***For*k∈Z, *let us denote*

$$C_{k}=\frac{2\pi }{\epsilon }\left(\frac{T_{s}}{T_{0}}-k\right).$$

*Then*, *the fixed points of the* 1-*dimensional map* (23) *can be computed analytically and*: 

• *If*$1+a^{2}+C_{k}^{2}<0$*for all*k∈Z, *the map* (23) *has no fixed points*.

• *If there exists*k∈Z*such that*$1+a^{2}+C_{k}^{2}<0$*and*

$$\left|\frac{-aC_{k}+\sqrt{1+a^{2}-C_{k}^{2}}}{1+a^{2}}\right|\leq 1,$$

*the map* (23) *has the fixed point*

$$\theta_{+}^{*}=\frac{1}{2\pi }\arccos\left(\frac{-aC_{k}+\sqrt{1+a^{2}-C_{k}^{2}}}{1+a^{2}}\right).$$

*Moreover*, *if*$a\leq C_{k}$*and*

$$\left|\frac{-aC_{k}-\sqrt{1+a^{2}-C_{k}^{2}}}{1+a^{2}}\right|\leq 1,$$

there exists also another fixed point

$$\theta_{-}^{*}=\frac{1}{2\pi }\arccos\left(\frac{-aC_{k}-\sqrt{1+a^{2}-C_{k}^{2}}}{1+a^{2}}\right).$$

*Proof* The fixed points $\theta^{*}$ of map (23) must satisfy

$$\epsilon \mathrm{PRC}\left(\theta^{*}\right)+\frac{T_{s}}{T_{0}}=k$$

for some k∈Z, or equivalently

$$\mathrm{PRC}\left(\theta^{*}\right)+\frac{C_{k}}{2\pi }=0.$$

Substituting $\mathrm{PRC}(\theta^{*})$ in the above equation by expression (31) with σ=0 and rearranging terms we have

(33) $$\sin\left(2\pi \theta^{*}\right)=a\cos\left(2\pi \theta^{*}\right)+C_{k}.$$

Taking squares of both sides of the equality and using trigonometric properties, we obtain

$$\left(1+a^{2}\right)\cos^{2}\left(2\pi \theta^{*}\right)+2aC_{k}\cos\left(2\pi \theta^{*}\right)+C_{k}^{2}-1=0,$$

which is an equation of degree 2 in $\cos(2\pi \theta^{*})$. Solving it, after some simplifications, we obtain

(34) $$\cos\left(2\pi \theta^{*}\right)=\frac{-aC_{k}\pm \sqrt{1+a^{2}-C_{k}^{2}}}{1+a^{2}}.$$

It is clear that for Eq. (34) to have real solutions, the right-hand side must have modulus at most 1 and $1+a^{2}-C_{k}\geq 0$. In this case, the solutions of (34) are

$$\theta_{\pm }^{*}=\frac{1}{2\pi }\arccos\left(\frac{-aC_{k}\pm \sqrt{1+a^{2}-C_{k}^{2}}}{1+a^{2}}\right).$$

However, as we have taken squares in Eq. (33), we still have to check whether $\theta_{+}^{*}$ and $\theta_{-}^{*}$ are solutions of (33). It is easy to check that $\theta_{+}^{*}$ always solves (33), while $\theta_{-}$ is a solution only when $a\leq C_{k}$. □

**Fig. 4:**
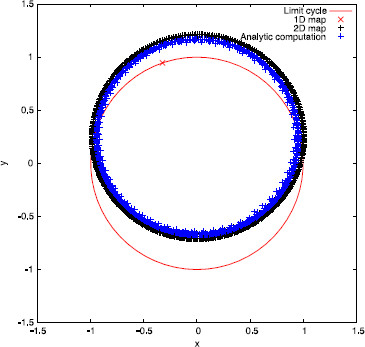
Comparison of iterative maps for periodic pulse-train stimuli. Sequences $K(\theta_{n},\sigma_{n})$, for n>200, computed using maps (22), (23) and (32) respectively

*Remark 4.3* A natural question is whether the 2-dimensional map (22) and the sequence (32) have the same qualitative behavior. As an example, let us take ϵ=0.03, α=0.1 and a=10. In this case, there exists just the fixed point $\theta_{+}^{*}$ for the 1-dimensional map (23). So, let us take the initial condition $\left(\theta_{0},\sigma_{0})=(\theta_{+}^{*},0\right)$ and compute its iterates by the three different maps (22), (23), and (32). In Fig. 4, we plot the sequences $K(\theta_{n},\sigma_{n})$ (for clarity, we have just plotted those with n>200). As one can see, map (23) fails to predict correctly the qualitative behavior of the solution, since (32) seems to be attracted to a quasi-periodic orbit and not a fixed point. On the contrary, map (22) correctly predicts this qualitative behavior. 

From now on, we will take the initial condition to be $\left(\theta_{0},\sigma_{0})=(0.8,0\right)$, that is, $\left(x_{0},y_{0})\approx (0.30901,-0.95106\right)$. In order to explore the effect of both the hyperbolicity and the transversality of the isochrons to the limit cycle, we will plot the different approximations of the rotation numbers *ρ*, $\rho_{2D}$, and $\rho_{1D}$ for different values of the parameters *a* and *α*.

**Fig. 5:**
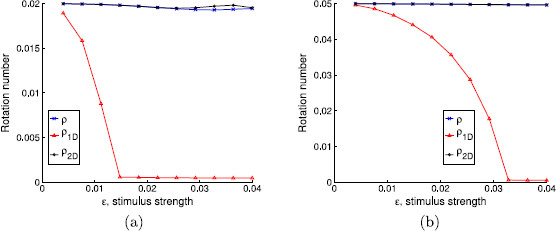
Rotation numbers as a function of the stimulus strength. Rotation numbers as a function of the stimulus strength for parameter values α=0.1 and a=10. Stimulation periods are **a**$T_{s}=0.0628319\approx T_{0}/50$, **b**$T_{s}=0.1570800\approx T_{0}/20$

First of all, we will take α=0.1 and a=10. This corresponds to considering a weakly hyperbolic limit cycle with isochrons that are almost tangent to it. In Fig. 5, we show the rotation numbers obtained for different amplitudes and for two different stimulus periods $T_{s}$. In this case, in order to make the rotation number $\rho_{1D}$ stabilize, we have taken N=1000. 

Observe also the agreement with the result in Lemma 4.2, which predicts the appearance of the fixed point of the 1D map when $1+a^{2}-C_{k}^{2}=0$, that is, when $C_{k}^{2}=101$ or, equivalently after substituting $T_{s}=T_{0}/m$, $\epsilon =2\pi /(\sqrt{101}m)$. The fixed point appears at ϵ≈0.0125 for m=50 (panel (a) in Fig. 5) and ϵ≈0.0312 for m=20 (panel (b) in Fig. 5); both values coincide with the downstroke of the corresponding values of $\rho_{1D}$.

**Fig. 6:**
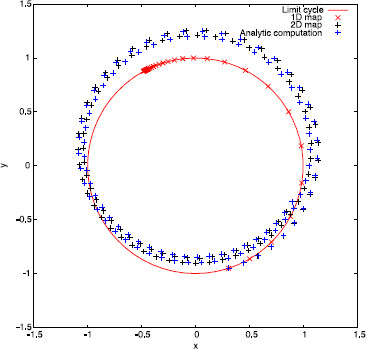
Iterates for a weakly hyperbolic limit cycle. First 100 iterates of sequences $K(\theta_{n},\sigma_{n})$ computed using the three different methods. Parameter values are taken to be α=0.1, a=10, ϵ=0.022 and $T_{s}=0.0628319\approx T_{0}/50$

One can see that the rotation number obtained with the 1-dimensional map diverges from the analytical computation, while the one obtained with the 2-dimensional map does not. This wrong prediction by the 1-dimensional approach is consistent for all intermediate values of $T_{s}$ (not shown here). We point out that, although the difference between the 1-dimensional approach and the other two seems rather small (it ranges from 10^−3^ to 10^−2^), we can identify a wrong prediction of the qualitative behavior of the system by the 1-dimensional map. Indeed, in the cases where $\rho_{1D}\approx 0$ but $\rho_{2D},\rho \neq 0$, the 1-dimensional map (23) has a fixed point, while the other two do not (see Remark 4.3). For example, in Fig. 6, we plot in the phase space the first 100 iterates of the sequences $K(\theta_{n},\sigma_{n})$, where $\left(\theta_{n},\sigma_{n}\right)$ are obtained, respectively, using the 2-dimensional map (22), the 1-dimensional map (23), and expression (32). While for the 1D map a fixed point is reached, for the 2D and the analytic approaches it seems that the dynamics are not so simple. Observe that this different qualitative behavior is obtained in spite of the initial condition being on the limit cycle. 

**Fig. 7:**
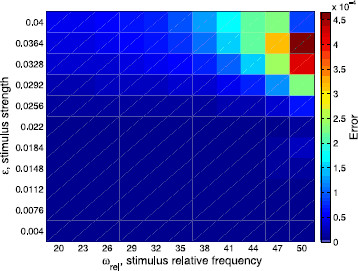
Differences among the 2D prediction and the analytic rotation numbers. Absolute difference between the rotation number obtained with the 2-dimensional approach and the analytic one, that is $\left|\rho_{2D}-\rho \right|$, in the two-parametric space $\left(\omega_{\mathrm{rel}},\epsilon \right)$

**Fig. 8:**
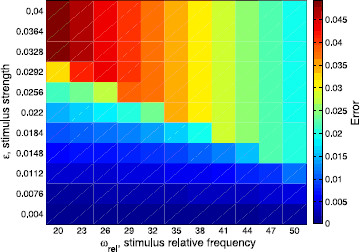
Differences among the 1D prediction and the analytic rotation numbers. Absolute difference between the rotation number obtained with the 1-dimensional approach (phase-reduction hypothesis) and the analytic one, that is $\left|\rho_{1D}-\rho \right|$, in the two-parametric space $\left(\omega_{\mathrm{rel}},\epsilon \right)$

**Fig. 9:**
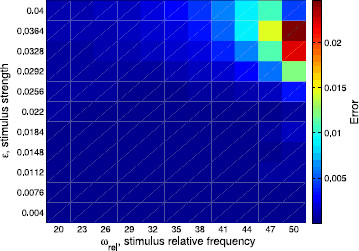
Ratio between errors given by the 2D prediction and by the 1D prediction. Ratio of the absolute difference between the 2-dimensional approach and the analytic one (numerator, see Fig. 7) over the absolute difference between the 1-dimensional approach and the analytic one (denominator, see Fig. 8), that is, $\left|\rho_{2D}-\rho |/|\rho_{1D}-\rho \right|$

Another visualization of the agreement or disagreement between the different approximations of the rotation numbers is provided in Figs. 7, 8, and 9. We show the differences between them depending on both *ϵ* (that is, the strength of the stimulus) and $\omega_{\mathrm{rel}}:=\omega_{s}/\omega_{0}=T_{0}/T_{s}$ (the ratio between the frequency of the stimulus and the frequency of the limit cycle). In Fig. 7, we plot the absolute difference between the rotation number obtained with the 2-dimensional approach and the analytic one, namely $\left|\rho_{2D}-\rho \right|$, whereas in Fig. 8, we plot the error when using the phase-reduction hypothesis, namely $\left|\rho_{1D}-\rho \right|$. Both errors are compared in Fig. 9, where the ratio $\left|\rho_{2D}-\rho |/|\rho_{1D}-\rho \right|$ is displayed. As expected, one can see in Figs. 7 and 8 that, fixing the stimulus period $T_{s}$, the worst approximations of *ρ* given respectively by $\rho_{2D}$ and $\rho_{1D}$ are obtained for high values of *ϵ*. However, fixing the strength of the stimulus *ϵ*, the results for both cases are different: while for the 2-dimensional map the worst results are for high frequency ratios $\omega_{\mathrm{rel}}$, for the 1D approach the worst results are obtained, in general, for low $\omega_{\mathrm{rel}}$. Finally, as we also expected, in Fig. 9 we can appreciate that the 2D approach is always better than the 1D. Moreover, the difference between $\rho_{2D}$ and *ρ* is, in the worst case, two orders of magnitude smaller than the difference between $\rho_{1D}$ and *ρ*. 

**Fig. 10:**
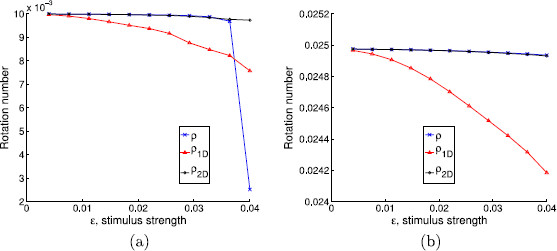
Effect of weak hyperbolicity and normal isochrons in the phase prediction. Rotation numbers for different stimulus strengths in case of weak hyperbolicity and normal isochrons (α=0.1 and a=0). Stimulation periods are **a**$T_{s}=0.0628319\approx T_{0}/50$, **b**$T_{s}=0.1570800\approx T_{0}/20$

**Fig. 11:**
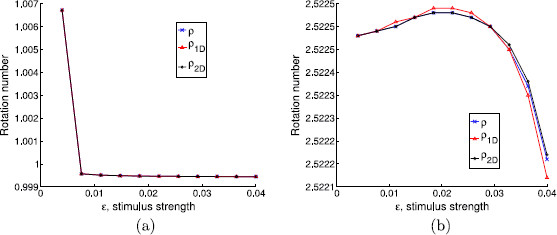
Effect of strong hyperbolicity and almost tangent isochrons in the phase prediction. Rotation numbers for different stimulus strengths in case of strong hyperbolicity and almost tangent isochrons (α=10 and a=10). Stimulation periods are **a**$T_{s}=0.0628319\approx T_{0}/50$, **b**$T_{s}=0.1570800\approx T_{0}/20$

**Fig. 12:**
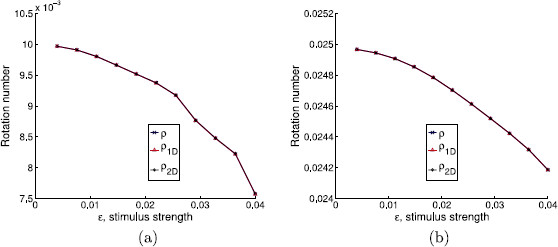
Effect of strong hyperbolicity and normal isochrons in the phase prediction. Rotation numbers for different stimulus strengths in case of strong hyperbolicity and normal isochrons (α=10 and a=0). Stimulation periods are **a**$T_{s}=0.0628319\approx T_{0}/50$, **b**$T_{s}=0.1570800\approx T_{0}/20$

As we mentioned above, we use this example to help us understanding the effect of the hyperbolicity of the limit cycle and the transversality of the isochrons to it in the validity of the PRC approach. In Figs. 10, 11, and 12, we plot the different approximations of the rotation numbers varying the parameters *α* (α=0 meaning loss of hyperbolicity) and *a* (a=0 meaning isochrons normal to the limit cycle). On one hand, when the limit cycle is strongly hyperbolic (for instance, α=10 as in Figs. 11 and 12), all approximations give a very similar result. Hence, in these two cases (even when the isochrons are almost tangent to the limit cycle, which corresponds to Fig. 11), the use of PRFs and ARFs instead of PRCs seems not necessary. In fact, that is what one can expect intuitively: if the attraction to the limit cycle is very strong, the system relaxes back to the asymptotic state very quickly, so that at each kick we can assume that the state variables are on the limit cycle. Of course, this will depend also on the frequency of stimulation $\omega_{s}$. 

On the other hand, in Fig. 10, where the contraction to the limit cycle is slow but the isochrons are almost orthogonal to the limit cycle, one can see that the 1D approach diverges from the 2D approach and the analytic one. However, for the range of *ϵ* and the two different stimulation periods $T_{s}$ (panels (a) and (b)) considered in Fig. 10, the 1D prediction still gives a fairly good approximation. Moreover, unlike the case where α=0.1 and a=10 (see Fig. 5), the 1D approach predicts a similar qualitative behavior as the other two approaches. The results for ϵ=0.04 in Fig. 10(a) raise another interesting question since the analytic rotation number *ρ* suddenly diverges from the 1D and the 2D rotation numbers. This is due to the fact that the iterates of the analytic map suddenly fail to encircle the critical point of the continuous system (located inside the limit cycle) while the iterates of the 1D and the 2D maps still do it. Thus, the rotation number for the analytic case may not give accurate information.

In conclusion, it seems that for the 2D map to represent a qualitative improvement with respect to the 1D it is necessary to have the combination of weak hyperbolicity of the limit cycle and “weak transversality” of isochrons to it. However, the role of hyperbolicity seems to be much more important, since in the presence of strong hyperbolicity the use of the 2D approach seems completely unnecessary, but for weak hyperbolicity the differences between the 1D and the 2D maps are present also when the isochrons are orthogonal to the limit cycle.

*Remark 4.4* Of course, considering a stimulus strength *ϵ* large enough, both maps (22) and (23) will not give correct predictions, since they are based on first-order approximations. In this case, one should consider PRFs of second (or higher) order to obtain a correct result; see, for instance, [23,24] for higher-order PRCs. One has to distinguish between these higher order response functions in terms of the stimulus strength from the second-order PRCs above mentioned (see [15] for instance) that relate to the second cycle after the stimulus. 

In the next example, we apply the same methodology to a more biologically inspired case: a conductance-based model for a point-neuron with two types of ionic channels.

#### 4.1.3 A Conductance-Based Model

We consider a reduced Hodgkin–Huxley-like system, with sodium and potassium currents, and only one gating variable:

(35) $$\begin{array}{rl} \dot{V} & =-\frac{1}{C_{m}}\left(g_{\mathrm{Na}}m_{\infty }(V)(V-V_{\mathrm{Na}})+g_{K}n(V-V_{K})+g_{L}(V-V_{L})-I_{\mathrm{app}}\right),\\ \dot{n} & =n_{\infty }(V)-n, \end{array}$$

where *V* represents the membrane potential, in mV, *n* is a nondimensional gating variable and the open-state probability functions are

$$\begin{array}{rcl} m_{\infty }(V) & = & \frac{1}{1+\exp(-(V-V_{\max,m})/k_{m})},\\ n_{\infty }(V) & = & \frac{1}{1+\exp(-(V-V_{\max,n})/k_{n})}. \end{array}$$

The parameters of the system are $C_{m}=1\;\mu {\text{F/cm}}^{2}$, $g_{\mathrm{Na}}=20{\text{ mS/cm}}^{2}$, $V_{\mathrm{Na}}=60\text{ mV}$, $g_{K}=10{\text{ mS/cm}}^{2}$, $V_{K}=-90\text{ mV}$, $g_{L}=8{\text{ mS/cm}}^{2}$, $v_{L}=-80\text{ mV}$, $V_{\max,m}=-20\text{ mV}$, $k_{m}=15$, $V_{\max,n}=-25\text{ mV}$, $k_{n}=5$.

**Fig. 13:**
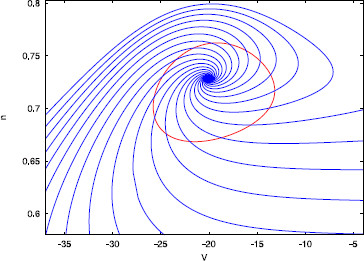
The limit cycle of the conductance-based model and its isochrons. The limit cycle (*red*) and equally spaced in phase isochrons (*blue*) for system (35) and $I_{\mathrm{app}}=190$

Here, we will take $I_{\mathrm{app}}=190\;\mu {\text{A/cm}}^{2}$. In this case, the system has a limit cycle with period $T_{0}\approx 1.3055442$, and its characteristic exponent is λ≈−0.6055956. That is, the limit cycle is weakly hyperbolic, and hence we expect that the 2-dimensional approach will give qualitatively different results with respect to the 1-dimensional approach. Figure 13 shows the limit cycle and its isochrons. 

**Fig. 14:**
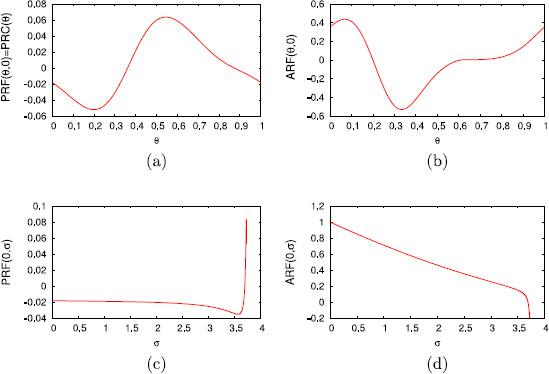
Phase and amplitude response functions along isochrons and *σ*-level sets. Phase response function (PRF) and amplitude response function (ARF) for system (35) and $I_{\mathrm{app}}=190$ on the limit cycle (σ=0), panels **a** and **b**, and for a specific isochron (θ=0), panels **c** and **d**

In Fig. 14, we show the PRF and the ARF on the limit cycle (σ=0), panels (a) and (b), and for a specific isochron (θ=0), panels (c) and (d). 

*Remark 4.5* We have chosen a value of the parameter $I_{\mathrm{app}}=190$ for which the system presents weak attraction to the limit cycle. However, for this value of $I_{\mathrm{app}}$, system (35) is not a model of a spiking neuron, but one with high voltage oscillations. Thus, this example is not intended to deal with a realistic setting of spike synchronization, but to illustrate how to deal with the tools introduced in this paper in the case where one does not explicitly have the parameterization *K*.

*Remark 4.6* In order to compute the parameterization *K* and the PRFs we have used the methods proposed in [8]. The same ideas can be applied to compute the ARFs. Briefly, the method consists of two steps. First, to compute the value of a given ARF near the limit cycle, where the numeric approximation of the parameterization *K* is valid, expression (16) is used. Second, to compute the value of some ARF far from the limit cycle, we just integrate the adjoint system (18) backwards in time using an initial condition for ARF close to the limit cycle.

**Fig. 15:**
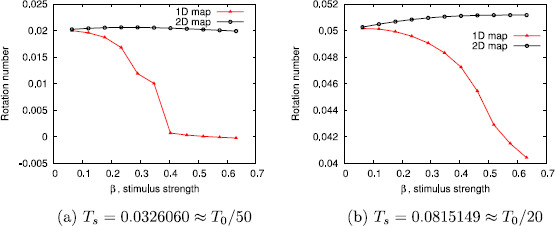
Rotation numbers for different stimulus strengths in the conductance-based model. Rotation numbers for different stimulus strengths and fixed stimulus periods for system (35)

**Fig. 16:**
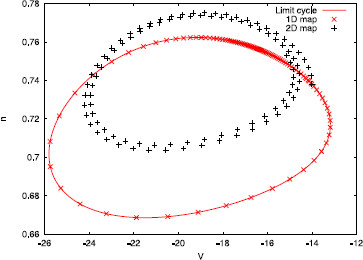
Iterates for a weakly hyperbolic limit cycle of the conductance-based model. Sequences $K(\theta_{n},\sigma_{n})$ computed using the 2-dimensional map (22) and the 1-dimensional map (23), respectively, for system (35). The strength of the stimulus is ϵ=0.574604, while the stimulation period is $T_{s}=0.026111\approx T_{0}/50$

Again, we have computed the rotation numbers as defined in (25) and (26) varying the strength of the stimulus *ϵ* with fixed stimulation periods. We have taken N=100 and initial conditions $\theta_{0}=0.089$ and $\sigma_{0}=0$. The results, for two different stimuli periods $T_{s}$, are shown in Fig. 15. Again, note that although the dynamics begin on the limit cycle (since $\sigma_{0}=0$), the behavior of the 1-dimensional approach and the 2-dimensional approach are quite different. Moreover, for ϵ>0.4 we find that $\rho_{1D}\approx 0$, while $\rho_{2D}\approx 0.02$. This can be interpreted, similarly to the previous example, as an indicator that the 1D map (23) has a fixed point, while the 2D map does not. Furthermore, this indicates that after 100 iterations of the 2D map (22), the state variables have turned approximately twice around the fixed point, as one can see from the plots of the sequences $K(\theta_{n},\sigma_{n})$ computed using both maps (see Fig. 16). 

## 5 Discussion

We have introduced general tools (the PRF and the ARF) to study the advance of both the phase and the amplitude variables for dynamical systems having a limit cycle attractor. These tools allow us to study variations of these variables under general perturbation hypotheses and extend the concept of infinitesimal PRCs which assumes the validity of the phase-reduction and is only true under strong hyperbolicity of the limit cycle or under weak perturbations. In fact, the PRFs and ARFs are first-order approximations of the actual variation of the phase and the amplitude, respectively, and so they are supposed to work mainly for weak perturbations; however, being an extension away from the limit cycle makes them more accurate than the PRCs even under strong perturbations. We thus claim that the phase-reduction has to be used with caution since assuming it by default may lead to completely wrong predictions in synchronization problems. We are not dismissing phase-reduction but trying to show the limits beyond which an extended scenario is required.

We have presented a computational analysis to understand the contribution of transient effects in first-order predictions of the phase response, focusing on the importance of the hyperbolicity of the limit cycle, but also on the relative positions of the isochrons with respect to the limit cycle.

In the examples studied, subject to pulse-train stimuli, we have compared the predictions obtained both with the new 2D map defined from the PRF and ARF and the 1D map defined from the classical PRC. Using rotation numbers, we have shown differences up to two orders of magnitude in favor of the 2D predictions, especially when the stimulation frequency is high or the stimulus is too strong. These results confirm previous numerical experiments with specific oscillators; see [22]. On the other hand, we have found that both weak hyperbolicity of the limit cycle and “weak transversality” of isochrons to it are important factors, although the role of hyperbolicity seems to be more crucial. In this paper, these achievements have been tested in a canonical model allowing comparisons with the exact solutions, and other numerical tests have been applied in a conductance-based model. The technique can be applied to other neuron models, and not necessarily for planar systems; *n*-dimensional systems would only require an additional computational difficulty in computing the associated (n−1) ARFs.

We would like to emphasize the importance of having good methods to compute isochrons (see [8-12]) since they are the cornerstone to study these transient phenomena that we have observed. They can be useful, not only for the problem illustrated here, but for other purposes like testing how far the experimentally recorded phase variations are from the theoretically predicted ones. In fact, they are the key concept to be able to predict the exact phase variation since, theoretically, if we know the parameterization *K* that gives the isochrons, the problem reduces to solving, at each step, (x,y)=K(θ,σ) and $\left(x^{\prime },y^{\prime })=K(\theta^{\prime },\sigma^{\prime }\right)$, where (x,y) is the point in the phase space where the pulse perturbation, *ϵ***w**, is applied and $(x^{\prime },y^{\prime })=(x,y)+\epsilon w$. Indeed, the PRFs and ARFs can be computed knowing only the first order in *K*; in principle, then they are valid only for weak perturbations, but easier to compute. Other refinements could be obtained by computing second order PRFs and ARFs by using the second-order approximations of the isochrons. Further extensions include also the possibility of computing response curves for long (in time) stimulus rather than pulsatile stimuli.

## Appendix:  The Vector Field for the A-Curves

We prove here that given an analytic local diffeomorphism *K*, as in (5), satisfying (6), the A-curves are the orbits of a vector field *Z*, satisfying [X,Z]=[Z,X]=0.

This is equivalent to proving that DXZ=DZX.

Taking derivatives with respect to *θ* in Eq. (6), we get

$$\left(\frac{1}{T}\partial_{\theta }+\frac{\lambda }{T}\sigma \partial_{\sigma }\right)\partial_{\theta }K=(DX\circ K)\partial_{\theta }K,$$

and using (14), we get

$$\left(\frac{1}{T}\partial_{\theta }+\frac{\lambda }{T}\sigma \partial_{\sigma }\right)(Z\circ K)=(DX\circ K)(Z\circ K).$$

By the chain rule,

$$(DZ\circ K)\left(\frac{1}{T}\partial_{\theta }+\frac{\lambda }{T}\sigma \partial_{\sigma }\right)K=(DX\circ K)(Z\circ K),$$

and again, by the invariance equation (6), we obtain

(36) (DX∘K)(Z∘K)=(DZ∘K)(X∘K).

as we wanted to prove.

## Competing Interests

The authors declare that they have no competing interests.

## Authors’ Contributions

The three authors have equally contributed.
